# Anti-Inflammatory Effects of a *Mytilus coruscus* α-d-Glucan (MP-A) in Activated Macrophage Cells via TLR4/NF-κB/MAPK Pathway Inhibition

**DOI:** 10.3390/md15090294

**Published:** 2017-09-20

**Authors:** Fuyan Liu, Xiaofeng Zhang, Yuqiu Li, Qixin Chen, Fei Liu, Xiqiang Zhu, Li Mei, Xinlei Song, Xia Liu, Zhigang Song, Jinhua Zhang, Wen Zhang, Peixue Ling, Fengshan Wang

**Affiliations:** 1 Shandong Academy of Pharmaceutical Science, Jinan 250101, China; chenqixin1010@aliyun.com (Q.C.); 15066696818@163.com (X.Z.); huoruhun@163.com (L.M.); ivyliu@yahoo.com (X.L.); zgangsong@163.com (Z.S.); 15098978003@163.com (J.Z.); 2 School of Pharmaceutical Sciences, Shandong University, Jinan 250012, China; xinleisongsxl@126.com; 3 School of Life Sciences, Lanzhou University, Lanzhou 730000, China; xfzhang2014@lzu.edu.cn; 4 Shandong University of Traditional Chinese Medicine, Jinan 250355, China; xiaoyusd2010@163.com; 5 School of Pharmacy, Second Military Medical University, Shanghai 200433, China; lfyflying@sohu.com

**Keywords:** THP-1 macrophages, anti-inflammatory, TLR4, NF-κB, MAPK, SPR analysis

## Abstract

The hard-shelled mussel (*Mytilus coruscus*) has been used as Chinese traditional medicine for thousands of years; however, to date the ingredients responsible for the various beneficial health outcomes attributed to *Mytilus coruscus* are still unclear. An α-d-Glucan, called MP-A, was isolated from *Mytilus coruscus*, and observed to exert anti-inflammatory activity in THP-1 human macrophage cells. Specifically, we showed that MP-A treatment inhibited the production of inflammatory markers, including TNF-α, NO, and PGE2, inducible NOS (iNOS), and cyclooxygenase-2 (COX-2), in LPS-activated THP-1 cells. It was also shown to enhance phagocytosis in the analyzed cells, but to severely inhibit the phosphorylation of mitogen-activated protein kinases (MAPKs) and the nuclear translocation of NF-κB P65. Finally, MP-A was found to exhibit a high binding affinity for the cell surface receptor TLR4, but a low affinity for TLR2 and dectin-1, via surface plasmon resonance (SPR) analysis. The study indicates that MP-A suppresses LPS-induced TNF-α, NO and PEG2 production via TLR4/NF-κB/MAPK pathway inhibition, and suggests that MP-A may be a promising therapeutic candidate for diseases associated with TNF-α, NO, and/or PEG2 overproduction.

## 1. Introduction

The hard-shelled mussel (*Mytilus coruscus*) is one of the main species of marine shellfish inhabiting broad regions of East Asian coastal areas, and has been used as both a food and medicine for thousands of years [1]. Many health benefits have been attributed to its use as a Chinese traditional medicine, including the nourishment of the liver and kidneys, the strengthening of the immune system, and the treatment of various diseases, such as goiter tumors, male impotence, and female menoxenia. In recent years, some biologically active polysaccharides [1], peptides [2,3], and lipid extracts [4] have been isolated from *Mytilus coruscus*, and evaluated to determine their pharmacological efficacy; nevertheless, to date, the ingredients responsible for the various beneficial health outcomes attributed to *Mytilus coruscus* are still unclear.

Polysaccharides isolated from natural sources can affect a variety of biological activities, such as immunity, suggesting their potential use as immunomodulatory agents with broad applications [5,6]. For example, a glycogen polysaccharide extracted from *Perna canaliculus* has been shown to exert anti-inflammatory activities [7], and similarly, a heparin-like substance isolated from the marine clams *Anomalocardia brasiliana*, has been shown to bind anti-thrombin III (ATIII) and to thereby exert a strong anticoagulant effect [8].

**Figure 1:**
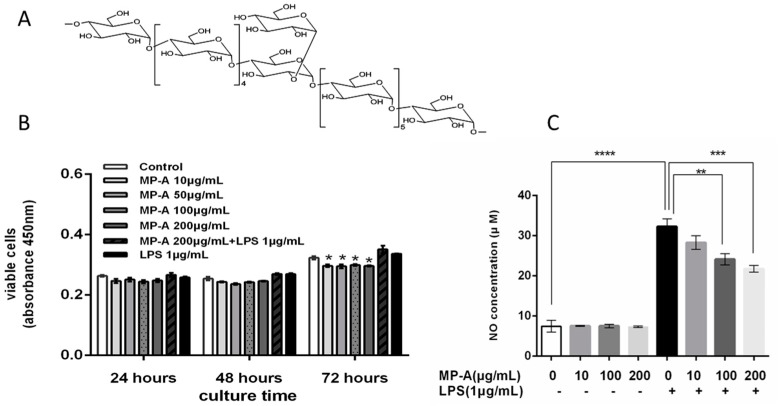
MP-A structure and effects of MP-A on cell viability and nitric oxide (NO) production of THP-1 cells with and without lipopolysaccharide (LPS) treatment. (**A**) MP-A structure; (**B**) Effects of 24, 48, and 72 h of MP-A and/or LPS treatment on THP-1 cell cytotoxicity. * *p* < 0.05 as compared to the control group (*n* = 3); (**C**) Effect of MP-A treatment on NO production in THP-1 cells with or without LPS. NO production was inferred from the level of nitrite formed in the supernatant, as detected using the Griess reagent. The absorbance of treated cells at 540 nm was measured against distilled water using the Griess reagent as a blank, and sodium nitrite as a standard sample. ** *p* < 0.01, *** *p* < 0.001, and **** *p* < 0.0001 compared with 1 μg/mL LPS treatment group (*n* = 3).

Various polysaccharides isolated from the hard-shelled mussel have received increasing attention in recent years. For example, the MP-1 polysaccharide from *Mytilus coruscus* has been demonstrated to exert a protective effect against acute liver injury [1], and the MEP polysaccharide from *Mytihus edulis* Linnaeus has been shown to both significantly ameliorate delayed-type hypersensitivity and phagocytosis, and to improve immune function in mice [9]. In our previous studies, we isolated the MA polysaccharide from *Mytilus coruscus*, and identified it to have anti-hyperlipidemic effects on experimental atherosclerosis in rabbits [10]. In the present study, we isolated a high-molecular-weight α-d-Glucan, named MP-A, from the hard-shelled mussel (*Mytilus coruscus*). We identified MP-A to contain repeating units of d-glucose, and to be structured such that the main chain was connected by α1-4 glucosidic bonds, and that a d-glucose was connected to this chain every twelve monosaccharides by α1-2 glucosidic bonds to form a branch. The molecular weight of MP-A was established to be approximately 1.2 × 10^3^ kDa, and the structure was shown in Figure 1A.

Glucans have been previously reported to exhibit significant bioactivity, and in particular, β-Glucans were extensively investigated between 1990 and 2000. These previous studies showed β-Glucans to exert anti-infective and anti-tumorigenic activity via the activation of leukocytes [11,12,13], and the production of reactive oxygen intermediates, inflammatory mediators such as NO, and TNF-α [11,14,15]. More recently, the α-glucans have begun to attract the attention of various research groups. For example, the α-glucan YCP, which is composed of α-d-(1–4)-linked glucose residues, has been recently revealed to inhibit tumor growth by modulating the innate and adaptive host immune responses to enhance macrophage activity, promote lymphocyte proliferation, and to induce cytokine secretion [16]. Similarly, six homogeneous, low-molecular-weight α-glucans (LMWYCP-1 to LMWYCP-6) have been shown to modulate the activity of toll-like receptors (TLRs), and thus, B lymphocytes [17].

Macrophages play a unique role in the immune system, in that they do not only elicit an innate immune response, but also are effector cells that counteract inflammation and infection. Furthermore, they are also critical to the maintenance of a functional interface between no-adaptive and adaptive immunity, and mediate various other functions, such as antigen processing and presentation to T cells.

THP-1 is a human leukemia monocytic cell line that has been widely used to study monocyte/macrophage function, signaling pathway mechanisms, and drug transport, and is commonly used to investigate the regulation of macrophage activity [18]. Lipopolysaccharide (LPS) is a major component of the outer membrane of gram-negative enteric bacteria [19]. During inflammatory processes, LPS induces the production of pro-inflammatory cytokines and small mediators, such as nitric oxide (NO), and PGE_2_ [20]. LPS-stimulated THP-1 macrophages have been shown to express the *MD2*, *CD14*, and *MyD88* genes that are required for LPS signaling in vivo [18]. When macrophages are activated by LPS, the TLR4 signaling pathway is initiated, leading to the phosphorylation of mitogen-activated protein kinase (MAPK), and the activation of the transcription factor nuclear factor-kappa B (NF-κB) [21], and the induction of pro-inflammatory factors including NO, inducible nitric oxide synthase (iNOS), interleukin-1β (IL-1β), interleukin-6 (IL-6), tumor necrosis factor (TNF)-α, and cyclooxygenase-2 (COX-2), etc. The MAPK family of proteins, including extracellular signal regulated kinase (ERK), c-Jun *N*-terminal kinase (JNK), and p38, regulate inflammatory and immune responses, and their respective signaling essential for LPS-induced iNOS and COX-2 expression in macrophages [22,23].

In the present study, LPS-induced THP-1 cells were used as an inflammatory model to investigate the effects of MP-A immunomodulation of THP-1 macrophages. To identify possible cell membrane receptors for MP-A, and putative molecular mechanisms underlying MP-A immunomodulation of THP-1 macrophages, the binding affinity of MP-A for TLR4 and/or TLR2 was assessed using surface plasmon resonance (SPR). To our knowledge, limited publications currently report on the immunomodulatory effects of combined treatment with LPS with MP-A. Thus, in the present study, we investigated the effects of MP-A on LPS-induced pro-inflammatory cytokine secretion by THP-1 cells, and on LPS-induced signal transduction.

## 2. Materials and Methods

### 2.1. Reagents and Antibodies

MP-A (purity 97.9% *w*/*w*) was produced and purified by Shandong Academy of Pharmaceutical Science (Jinan, China), and verified to have an average molecular weight (*M*w) of 1200 kDa via a size exclusion chromatography multi-angle laser scattering (SEC-MALLS) system (Wyatt Technology Corp, Santa Barbara, CA, USA), the preparation and structural characterization of MP-A has been introduced in other paper. LPS from *Escherichia coli* O55:B5, phorbol-12-myristate-13-acetate (PMA), and FITC-dextran (FD40S) were purchased from Sigma–Aldrich Chemical Co. (St. Louis, MO, USA) and the average MW of Dextran was 40,000 Da. Recombinant human Dectin-1, TLR4, and TLR2 were purchased from R&D Systems (Minneapolis, MN, USA). Fetal bovine serum (FBS), and other cell culture reagents were purchased from Gibco BRL Co. (Grand Island, NY, USA). The Cell Counting Kit-8 (CCK-8) Assay Kit was obtained from Beyotime (Wuhan, China). Penicillin and streptomycin were purchased from HyClone (Logan, UT, USA). ELISA kits for PGE2, and TNF-α were purchased from Biolegend (San Diego, CA, USA). The p-p38, p38, p-JNK1/2, JNK1/2, p-ERK1/2, ERK1/2, p-P65, P65, COX-2, and iNOS antibodies were obtained from Abcam (Cambridge, UK). The β-actin monoclonal antibodies were obtained from Cell Signaling Technology (Beverly, MA, USA). NF-κB Activation-Nuclear Translocation Assay kits were purchased from Beyotime Institute of Biotechnology (Haimen, China). All of the other chemicals were of analytical grade. The horseradish peroxidase (HRP)-conjugated goat anti-rabbit IgG secondary antibody was purchased from Santa Cruz Biotechnology Inc. (Santa Cruz, CA, USA).

### 2.2. THP-1 Cell Culture

THP-1, a human leukemia monocytic cell line extensively used to study the modulation of monocytes and macrophages, was purchased from the Shanghai Cell Bank, the Institute of Cell Biology, China Academy of Sciences (Shanghai, China). THP-1 cells in the monocyte state can be differentiated into a macrophage-like phenotype via stimulation with phorbol-12-myristate-13-acetate (PMA) (50 ng/mL, 24 h). The cells were cultivated in RPMI 1640 medium (containing 10% heat-inactivated FBS, and 1% penicillin/streptomycin), at 37 °C in a humidified atmosphere (5% CO_2_, 95% air).

### 2.3. Cell Viability Assay

The viability of THP-1 cells was determined using the Cell Counting Kit-8 (CCK-8) Assay Kit (Beyotime, Wuhan, China). The cells were primed for differentiation with 50 ng/mL PMA for 24 h [24], and then seeded on 96-well plates at a density of 5 × 10^4^ cells/mL, in 100 μL medium, and left to incubate overnight. They were then treated with MP-A (10, 50, 100, or 200 μg/mL dissolved in serum-free medium), and/or LPS (1 μg/mL dissolved in serum-free medium) for 24, 48, or 72 h (37 °C, 5% CO_2_). CCK-8 (10 µL) was added to each well 4 h prior to culture termination, and the optical density of each well was measured (450 nm) using a microplate reader (Infinite M200 PRO, TECAN, Männedorf, Switzerland).

### 2.4. Determination of Phagocytic Uptake of FITC-Labeled Dextran

The cells were primed for differentiation with 50 ng/mL PMA for 24 h [24], and then seeded on 24-well plates at a density of 5 × 10^4^ cells/mL in 1 mL medium and left to incubate overnight. They were then treated with MP-A (10, 100 or 200 μg/mL dissolved in serum-free medium), and/or LPS (1 μg/mL dissolved in serum-free medium) for 24 h. Then THP-1 cells were collected, resuspended, and incubated in 100 μL FITC-labeled dextran (1 mg/mL, 37 °C, 30 min). Cells incubated with FITC-labeled dextran only (4 °C, 30 min) were used as a control and cells incubated without FITC-labeled dextran were used as a blank control. After incubation, ice-cold PBS (2 mL, containing 2% FBS) was added to the cells to inhibit phagocytosis, and they were then washed three times (ice-cold PBS). Cellular uptake of FITC-labeled dextran was analyzed by flow cytometry (BD FACSAria ™ III).

### 2.5. ELISA for Cytokine Estimation

TNF-α and PGE_2_ were measured by using a commercial ELISA kit according to the manufacturers’ instructions. THP-1 cells were primed with PMA and seeded in a 96-well plate (density 1 × 10^6^ cells/mL), and cultured overnight. They were then treated first with MP-A (10, 50, 100, or 200 μg/mL, 1 h), and secondly with LPS (1 μg/mL, 6 or 24 h to enable the measurement of TNF-α or PGE_2_, respectively. Cell-free supernatants were finally collected and analyzed to measure the levels of both cytokines.

### 2.6. Nitric Oxide (NO) Production

The total nitrite content in the cell culture supernatant was measured using the Griess reagent. An equal volume of Griess reagent was added to the respective samples, and they were then incubated (30 min, 37 °C). The nitrite concentration was estimated by measuring the absorbance of samples at 545 and 630 nm (wavelength correction) against a sodium nitrite standard using an ELISA plate reader (Infinite M200 PRO, TECAN, Männedorf, Switzerland).

### 2.7. Western Blot Analyses

THP-1 cells were primed with PMA, seeded in a 100-mm culture dish (density 1 × 10^6^ cells/mL), cultured overnight, and then treated with MP-A (10, 50, 100, or 200 μg/mL) and LPS (1 μg/mL), and the cells were then incubated (37 °C) for 24 h. Cytosolic proteins were isolated using the Cytosol Fractionation Kit, according to the manufacturer's instructions. Total protein was measured by the bicinchoninic acid assay (BCA assay), and proteins were separated by SDS-PAGE and electro-transferred onto a polyvinylidene difluoride (PVDF) membrane. The PVDF membrane blots were blocked (1 h) using 8% non-fat powdered milk in TBST buffer, and then incubated overnight at 4 °C with appropriate primary antibodies against p-p38, p38, p-JNK1/2, JNK1/2, p-ERK1/2, ERK1/2, iNOS, COX-2, P65, and/or p-P65. After three washes (TBST buffer, 5 min), the PVDF membranes were incubated (2 h) with a goat anti-rabbit IgG, or goat anti-mouse IgG second antibody. After a final three washes (TBST buffer, 5 min), specific proteins were detected using an enhanced chemiluminescence kit (ECL, Millipore, Billerica, MA, USA) according to the manufacturer’s instructions. β-actin was used as a loading control.

### 2.8. Detection of NF-κB Activation and Nuclear Translocation

The detection of NF-κB nuclear translocation was carried out following the instruction of the kit (Beyotime Institute of Biotechnology, Haimen, China) [25,26].THP-1 cells were primed with PMA and seeded on glass coverslips in a 24-well plate (density 1 × 10^6^ cells/mL), cultured overnight, and then treated with 200 μg/mL MP-A with or without 1 μg/mL LPS treatments for 2 h. Then, cells grown on coverslips were fixed and blocked, and incubated overnight with the primary antibody against the p65 subunit of NF-κB at 4 °C, then washed in PBS, and incubated with Cy3-labeled secondary antibody for 2 h at room temperature. Finally, the cells were stained with 2 μM 2-(4-amidinophenyl)-6-indolecarbamidine dihydrochloride (DAPI) solution for 5 min. Each step above was followed by washing with ice-cold PBS three times for 5 min each. Then, the activation and nuclear translocation of NF-κB were observed using a laser scanning confocal microscope (LSM 780, Zeiss, Oberkochen, Germany).

### 2.9. Affinity Assay for MP-A with TLR4, TLR2 and Dectin-1

The affinity of MP-A for recombinant human TLR4, TLR2, and Dectin-1 was examined via SPR, and performed using a BIAcore 3000 (GE Healthcare, Uppsala, Sweden) according to the manufacturer’s instructions. Prior to analysis, recombinant human TLR4, TLR2, and Dectin-1 proteins (R&D Systems) were each immobilized on a CM5 sensor chip using an amine coupling kit. The surface of the chip was activated by EDC/NHS, and then TLR4, TLR2, and/or Dectin-1 (25 μg/mL) suspended in 10 mmol/L sodium acetate solution (pH 4.5) was flowed over it. When the ligand density reached the target resonance unit (RU) level, remaining active esters were quenched with a 1 mol/L ethanolamine solution (pH 8.5). (Control flow cells received identical treatment, but no TLR4, TLR2, and/or Dectin-1 protein was added to the 10 mmol/L sodium acetate solution). The resultant sensor chip was stored in 10 mM phosphate-buffered saline as a running buffer, and maintained at 25 °C for all of the experiments. MP-A (dissolved in running buffer at a series of concentrations) was injected into the channels, and resulting signals were detected using the BIAcore program (GE). The sensor chip was regenerated in a 10 mM NaOH solution after each analysis. Affinity constants were calculated using BIA evaluation 4.1 software, by globally fitting the association and dissociation phases of overlay plots to a 1:1 Langmuir binding model.

### 2.10. Statistical Analyses

Multiple comparisons were statistically assessed using a one-way ANOVA, followed by a Tukey’s multiple comparison test. Results were expressed as the mean ± SEM, and a *p*-value of <0.05 was considered to indicate statistical significance.

## 3. Results

### 3.1. Effects of MP-A on THP-1 Cell Viability

Treatment of THP-1 cells with up to 200 μg/mL of MP-A over 48 h did not result in any significant cytotoxic effect; however, 72 h of MP-A treatment induced a slight decrease in cell viability (Figure 1B). LPS treatment (1 μg/mL), alone over a 24-h period, was insufficient to cause a significant increase in cell viability, as compared with the negative control (*p* > 0.05). We therefore studied the effects of MP-A on phagocytosis, and on NO and cytokine production, using a 24-h culture model that was unaffected by changes in cell quantity.

### 3.2. Effect of MP-A on NO Release by THP-1 Cells

LPS stimulation resulted in a significant increase in NO production (nitrite 31.34 ± 1.84 μM) relative to that in of unstimulated controls (nitrite 7.46 ± 1.38 μM) (Figure 1C). MP-A treatment dose-dependently inhibited NO production in LPS-stimulated THP-1 cells, such that approximately 33% inhibition was observed at an MP-A concentration of 200 μg/mL (Figure 1C). In contrast, NO production was not significantly affected by MP-A treatment alone when compared to the negative control group (*p* > 0.05).

### 3.3. Effect of MP-A on Phagocytic Uptake of FITC-Labeled Dextran by THP-1 Cells

**Figure 2:**
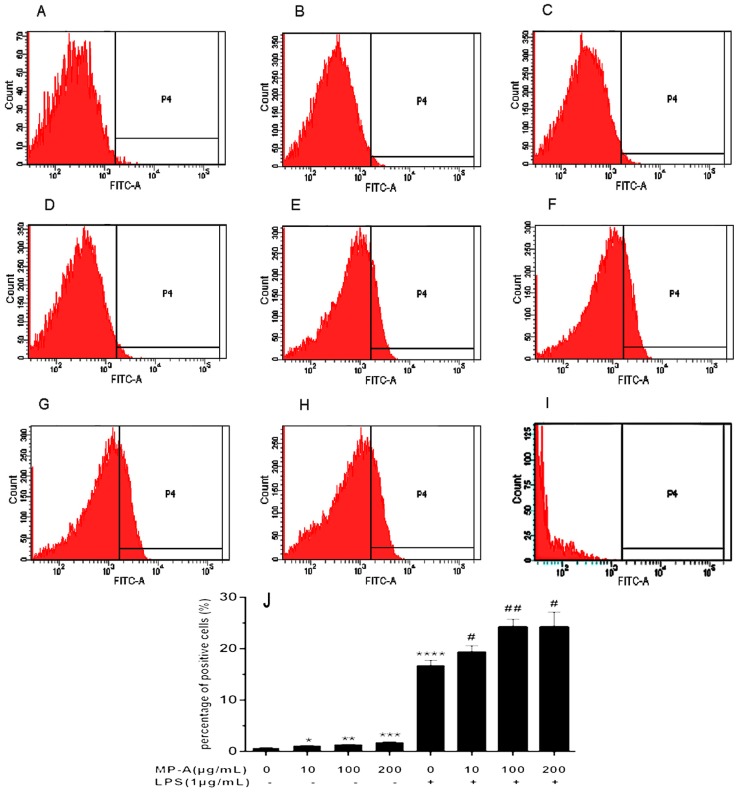
Effect of MP-A on THP-1 cell phagocytosis. (**A**–**H**) Representative fluorescence activated cell sorter (FACS) plots (three independent experiments), showing phagocytosis of THP-1 cells treated with (**A**) vehicle only (control); (**B**) 10 μg/mL MP-A; (**C**) 100 μg/mL MP-A; (**D**) 200 μg/mL MP-A; (**E**) 1 μg/mL LPS; (**F**) 1 μg/mL LPS + 10 μg/mL MP-A; (**G**) 1 μg/mL LPS + 100 μg/mL MP-A; and (**H**) 1 μg/mL LPS + 200 μg/mL MP-A; (**I**) blank control (**J**) Statistical analysis of results shown in (**A**–**H**). * *p* < 0.05, ** *p* < 0.01, *** *p* < 0.001, and **** *p* < 0.0001 compared to control (MP-A) group; ^#^ *p* < 0.05, and ^##^ *p* < 0.01, when compared to LPS group (*n* = 3).

The Phagocytic Uptake of FITC-labeled Dextran by THP-1 cells was analyzed using flow cytometry. The results of this analysis showed that MP-A significantly increased the capacity of cell phagocytic uptake of FITC labeled dextran in a dose-dependent manner at concentrations as low as 10 μg/mL, as compared to controls (e.g., phagocytic uptake of FITC labeled dextran was 1.13 ± 0.06% and 0.73 ± 0.15% in MP-A-treated and control cells, respectively, *p* < 0.05, *n* = 3; Figure 2A–D). LPS treatment also markedly stimulated THP-1 cell phagocytosis (e.g., phagocytic uptake of FITC labeled dextran was 16.73 ± 1.01% and 0.73 ± 0.15% in LPS-treated and control cells, respectively, *p* < 0.0001, *n* = 3; Figure 2A,E), and combined LPS and MP-A treatment further stimulated phagocytosis in a dose-dependent manner (Figure 2F–H).

### 3.4. MP-A Inhibition of TNF-α and PGE_2_ Release by LPS-Stimulated THP-1 Cells

**Figure 3:**
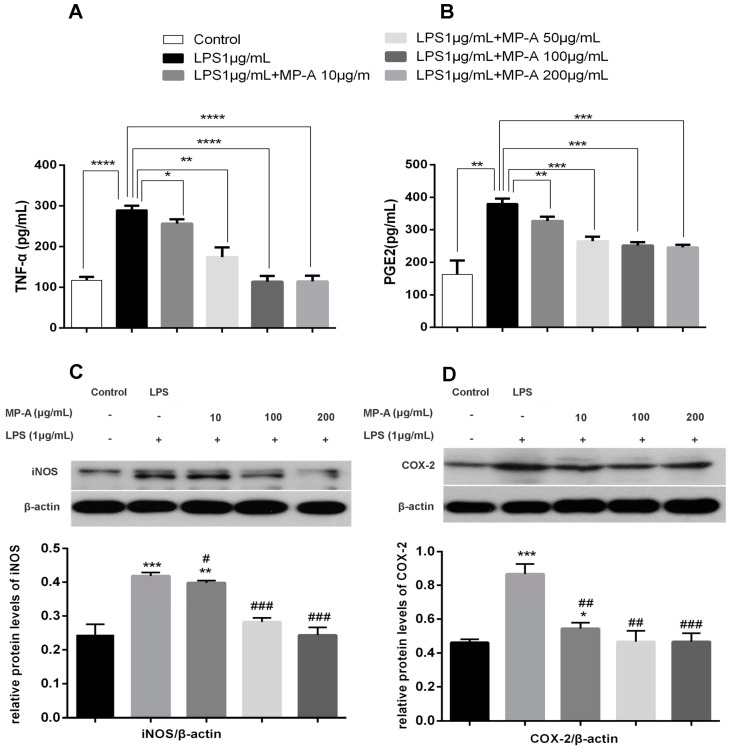
Effect of MP-A on TNF-α and PGE2 production and iNOS and COX-2 protein expression in LPS-stimulated THP-1 cells. (**A**,**B**) MP-A inhibition of TNF-α and PGE2 production in LPS-stimulated THP-1 cells. TNF-α (6-h stimulation) and PGE2 (24-h stimulation) were measured via an ELISA. The data represent three independent experiments. * *p* < 0.05, ** *p* < 0.01, *** *p* < 0.001, and **** *p* < 0.0001 compared to the LPS group. (**C**,**D**) MP-A inhibition of iNOS and COX-2 protein expression, as assessed by western blotting analysis in LPS-stimulated THP-1 cells. * *p* < 0.05, ** *p* < 0.01, and *** *p* < 0.001, when compared to controls; ^#^ *p* < 0.05, ^##^ *p* < 0.05, and ^###^ *p* < 0.001 compared to the LPS group (*n* = 3).

When compared with control cells, LPS treatment significantly increased TNF-α and PGE_2_ production in THP-1 cells. In contrast, MP-A treatment suppressed TNF-α production such that 60% inhibition was achieved by treating cells with 200 μg/mL MP-A for 6 h. Similarly, 24 h of MP-A treatment (200 μg/mL) resulted in a 35%-inhibition of PGE_2_ production (Figure 3A,B).

### 3.5. MP-A Inhibits iNOS and COX-2 Expression in LPS-Stimulated THP-1 Cells

A western blot analysis was conducted to investigate the effect of MP-A treatment on iNOS and COX-2 expression in LPS-stimulated THP-1 macrophages. As depicted in Figure 3C,D, COX-2 and iNOS protein expression were both dramatically increased by treatment with LPS alone compared to control group, whereas pre-treatment with MP-A decreased protein levels of COX-2 and iNOS in a dose-dependent manner. The reduction of iNOS and COX-2 protein expression was consistent with the above descried observed inhibition of NO and PGE_2_ (Figure 1C and Figure 3B).

### 3.6. MP-A Treatment Inhibits LPS-Induced Phosphorylation of MAPKs and NF-κB in THP-1 Cells

**Figure 4:**
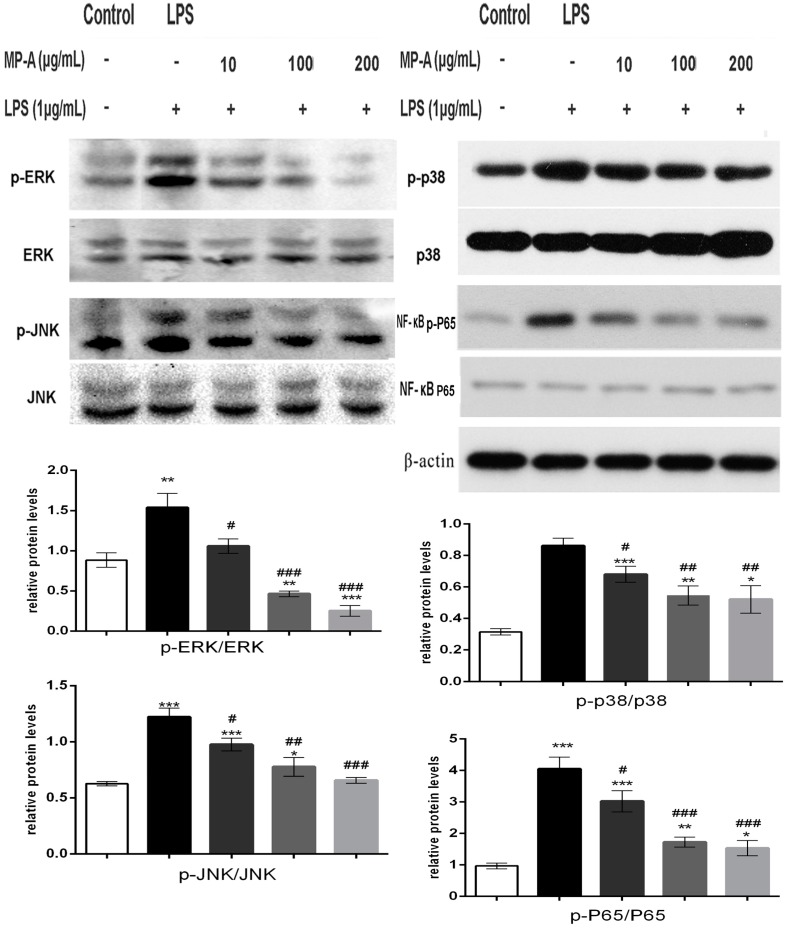
MP-A significantly attenuated LPS-induced activation of the mitogen-activated protein kinases (MAPK) and NF-κB signaling pathways in THP-1 cells. The ratios of p-p38/p38, p-JNK/JNK, p-ERK1/2/ERK1/2, and p-P65 /P65 were analyzed by western blot analysis. * *p* < 0.05, ** *p* < 0.01, *** *p* < 0.001, and **** *p* < 0.0001, when compared to controls; ^#^ *p* < 0.05, ^##^ *p* < 0.05, ^###^ *p* < 0.001, and ^####^ *p* < 0.0001 compared to the LPS group (*n* = 3).

LPS from Gram-negative bacteria is known to stimulate inflammatory responses via TLR4-initiation of several distinct signaling pathways, including the MAPK and NF-κB pathways. To determine whether either of these pathways was affected by MP-A treatment, cytoplasmic cell lysates were analyzed by western blotting with specific phosphor-antibodies against P65 (NF-κB), ERK1/2, JNK1/2, and p38 (MAPK). The results of this analysis showed MP-A to dose-dependently inhibit the LPS-induced phosphorylation of P65, ERK1/2, JNK1/2, and p38 in the THP-1 cells (Figure 4).

### 3.7. MP-A Prevented LPS-Induced Activation and the Nuclear Translocation of NF-κB in THP-1 Cells

**Figure 5:**
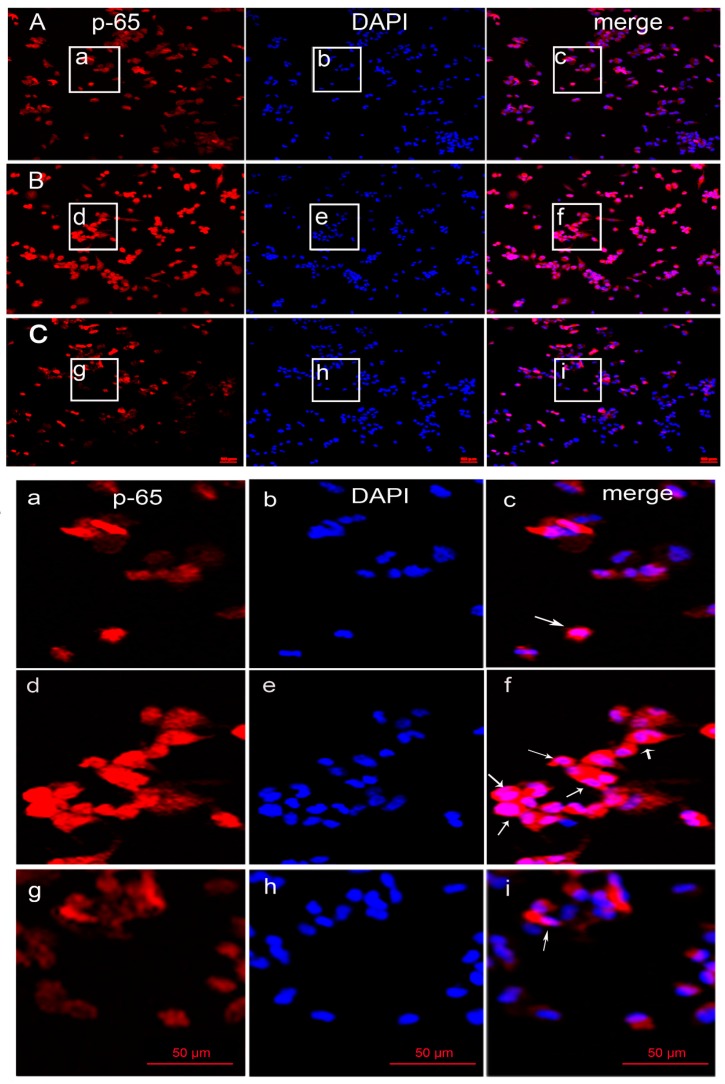
MP-A prevented LPS-induced activation and the nuclear translocation of NF-κB p65. THP-1 cells labeled with NF-κB subunit p65 (red) and DAPI (blue) were imaged simultaneously and merged (pink). (**A**) Control cells were treated with neither MP-A nor LPS solution. (**B**) Cells were treated with 1 μg/mL LPS for 24 h (**C**) Cells were treated with 200 μg/mL MP-A and 1 μg/mL LPS for 2 h. (a–i) Magnification imaging corresponding to the white boxes in Figure 5A–C. The images were obtained by confocal laser microscopy and overlay; the pink fluorescence (as white arrows show above) indicates location of p65 protein in nuclei.

NF-κB regulates many molecules of the various stages of the inflammatory response. In unstimulated control cells, NF-κB retains its original state as an inactive cytoplasmic complex by its inhibitor IκB. Upon LPS stimulation, the phosphorylation and degradation of IκB release NF-κB for nuclear translocation. We studied the NF-κB translocation using a primary antibody against the p65 subunit of NF-κB and the nuclei dye DAPI (Figure 5). When comparing with the control (Figure 5A(a–c)), NF-κB p65 traveled from the cytosol to the nucleus in macrophages by 1 μg/mL LPS treatment (Figure 5B(d–f)), however, 200 μg/mL MP-A obviously prevented LPS-induced activation and the nuclear translocation of p65 (Figure 5C(g–i)).

### 3.8. Affinity of MP-A for Recombinant Human TLR4, TLR2, and Dectin-1

**Figure 6:**
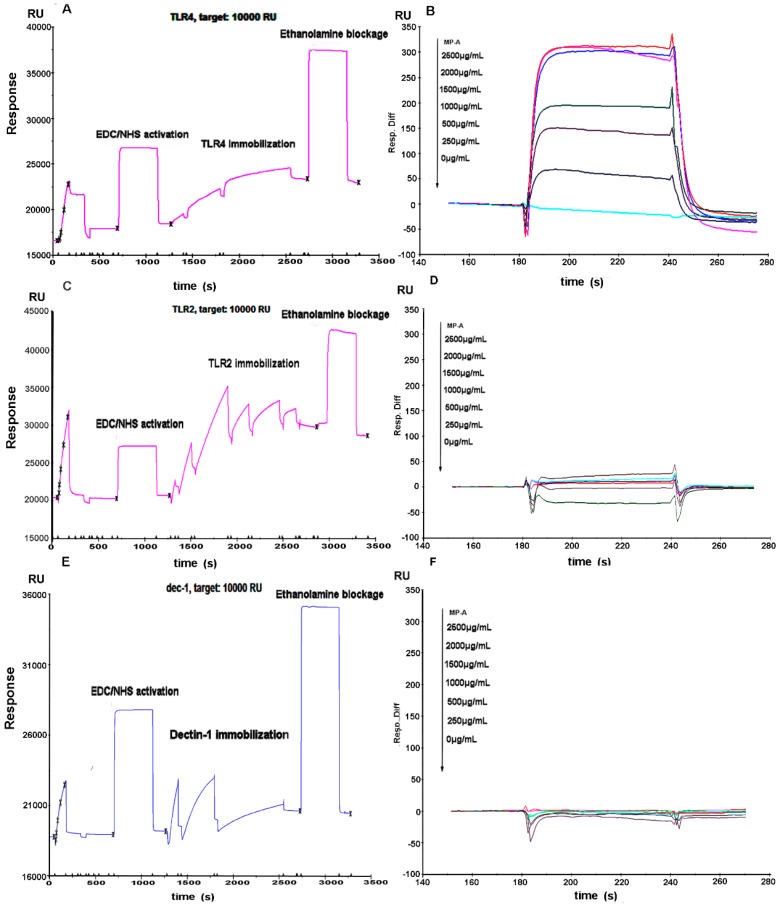
Binding affinity of MP-A for TLR4, TLR2, and Dectin-1, as evaluated by surface plasmon resonance (SPR) analysis. Cumulative MP-A concentrations were injected at 30 μL/min over 60-second association periods, with the concentrations shown next to the arrows. (**A**,**C**,**E**) TLR4, TLR2, and Dectin-1 immobilization on CM5 sensor chips, respectively. (**B**,**D**,**F**) Kinetic-binding responses of MP-A to TLR4, TLR2, and Dectin-1, respectively.

SPR technology (with a BIAcore 3000 biosensor system) was applied to analyze the affinity of MP-A for recombinant human TLR4, TLR2, and Dectin-1 proteins. Each of the proteins was immobilized on a CM5 chip (Figure 6A,C,E). The RU for TLR4, TLR2, and Dectin-1 bound to the CM5 chip surface was, respectively, 5022.4, 8333.8, and 1131.7. The sensorgrams of MP-A for TLR4, TLR2, and Dectin-1 demonstrated that MP-A exhibited a high binding affinity for TLR4 (KD = 5.07 × 10^−5^ M), but hardly any affinity for TLR2 and Dectin-1 even up to a concentration of 2500 μg/mL (Figure 6B,D,F).

## 4. Discussion

The preset study used THP-1 macrophages as an in vitro cell model to investigate the effect of MP-A on immune modulation. Given that LPS has been previously shown to activate multiple signaling pathways in macrophages, and to thereby induce the production of pro-inflammatory mediators and cytokines such as NO, TNF-α, and interleukins, we chose LPS-stimulation as the method for inducing experimental inflammation of the THP-1 cells. The results of the present study demonstrate MP-A to exert an anti-inflammatory effect in LPS-stimulated THP-1 cells, via the inhibition of the TLR4/NFκB and MAPK pathways.

An increase in phagocytic activity is one of the most characteristic features of macrophage activation, and the phagocytosis and subsequent destruction of microorganisms is the most important function of monocytes and macrophages in maintaining the immune response against infection. LPS has been previously reported to suppress the phagocytosis of immunoglobulin G-opsonized sheep red blood cells (SRBCs) by peritoneal macrophages. LPS suppresses phagocytosis of SRBCs by altering the distribution of microfilaments and microtubules, and this suppression is independent of cytokine (IL-1, IL-6, TNF- or IFN α/β) production [27,28]. LPS has been reported to increase obviously the phagocytic activity in RAW264.7 macrophages [29,30]. The data generated in the present study suggest that LPS treatment markedly increased phagocytic activity in THP-1 macrophage cells, and furthermore, these effects were synergistically increased by combined treatment with LPS and MP-A. While the molecular mechanism underlying the increased phagocytosis induced by MP-A is unknown, we hypothesize that it is likely to be independent of cytokine production in a similar manner to LPS, since cytokine production was observed to be reduced by MP-A treatment in LPS-simulated THP-1 cells.

NO is a key molecule in immune function, and has been shown to exert beneficial biological effects in a variety of cell types involved in immunomodulatory, inflammatory, and/or other physiological processes [31,32]. NO is synthesized by a family of enzymes known as NOS, comprising three main isoforms, constitutive NOS (nNOS), endothelial constitutive NOS (eNOS), and inducible NOS (iNOS). Of these, iNOS is the pro-inflammatory enzyme that is most critical for upregulating the levels of NO production in response to pro-inflammatory stimuli. For example, LPS macrophage stimulation induces NO production via iNOS [33,34]. In the present study, MP-A treatment was shown to effectively inhibit NO production in LPS-activated THP-1 macrophages via the inhibition of iNOS protein expression, without affecting cell viability. In contrast, MP-A treatment alone was found to be insufficient to affect NO production. 

Cyclooxygenase-1 (COX-1) and Cyclooxygenase-2 (COX-2) can regulate the production of prostaglandins [35]. COX-2 mediates the conversion of arachidonic acid to prostaglandin H2, a precursor of PGE_2_, in activated macrophages. Overexpression of iNOS and COX-2 has been previously found to lead to the overproduction of NO and PGE_2_, and this results in the development of inflammatory diseases. The results of the present study suggest that MP-A significantly suppresses COX-2 expression in LPS-stimulated THP-1 macrophages. Since one of the current strategies to mitigate inflammatory disorders is the modulation of iNOS and COX-2 expression, MP-A may be a promising potential therapeutic candidate for controlling inflammatory mediator overproduction.

TNF-α has been reported to trigger the downstream activation of inflammatory gene expression, and to cause rheumatoid arthritis, inflammatory bowel disease, psoriasis, and refractory asthma [36,37,38,39]. The present study demonstrated MP-A treatment of THP-1 cells to significantly attenuate TNF-α production in a dose-dependent manner. We previously observed a similar phenomenon to occur while treating RAW264.7 cells with curdlan sulfate (CS3) [29]. In the previous study, treatment with CS3 or curdlan alone was found to stimulate TNF-α production, whereas treatment of LPS-stimulated RAW264.7 cells with CS3 or curdlan instead attenuated TNF-α production to some extent. Another β-Glucan, LNT-S, that was isolated from *Lentinus edodes*, has also been determined to suppress NO and TNF-α production in LPS-stimulated RAW264.7 cells [15]. Interestingly, CS3, curdlan, and LNT-S have a β-glucan structure, while MP-A instead exhibits an α-glucan structure. Nevertheless, the results of the present study indicate that MP-A is similar to those β-glucans, and is a promising potential therapeutic agent for use in controlling diseases associated with pro-inflammatory mediator overproduction. β-glucans such as CS3 and LNT-S have been identified to bind the key macrophage surface receptor, Dectin-1 [15,28,29]. This receptor has been documented to be required for β-glucan recognition, and can collaborate with TLR2 to mediate the biological effects of β-glucans [40,41]. To determine whether MP-A binds to TLR2, TLR4, and/or Dectin-1 receptors, we evaluated potential interactions between MP-A and each of the three receptors using an SPR analysis. The results of this analysis show that MP-A can strongly bind TLR4, but not TLR2 nor Dectin-1, thus, TLR4, but not TLR2 or Dectin-1, can recognize the water-soluble α-glucan, MP-A.

TLR4 alone is not sufficient for LPS signaling, and indeed a secretory protein, MD-2, has also been shown to be an essential to this process [42]. TLR4 recognizes LPS in conjunction with MD-2 and CD14, such that they have been shown to form a physical complex on LPS-responsive cell surfaces [43,44]. We recently demonstrated LPS to exhibit a poor binding affinity for recombinant TLR4 using SPR technology, and provided more evidence for LPS signaling via TLR4 as a receptor is required for an additional molecule [30].

Various inflammatory diseases upregulate pro-inflammatory cytokines (such as TNF-α and IL-1β) and inflammatory mediators (such as NO and PGE_2_) via the NF-κB and MAPKs signaling pathways in macrophages [45,46]. The present study aimed to elucidate the way in which MP-A interacts in these processes. The results show that MP-A can prevent LPS-induced activation via regulating the phosphorylation of MAP kinase and/or NF-κB p65 signaling pathway components (Figure 4 and Figure 5). Specifically, MP-A inhibited LPS-induced iNOS and COX-2 expression, and subsequent NO and PGE_2_ production, via the selective suppression of the phosphorylation of ERK1/2, JNK, and p38 in the MAPK signaling pathway, and the nuclear translocation of P65 in the NF-κB signaling pathway. The mechanism by which MP-A exerts this anti-inflammatory activity in LPS-stimulated THP-1 cells is likely due to competitive binding to TLR4 at the cell membrane, leading to the inhibition of the LPS-TLR4 signaling pathway, and thus inhibiting the downstream phosphorylation of MAPK and the nuclear translocation of NF-κB pathway components. Other molecules involved in the inhibition mechanism will be further studied in detail in our future work.

## 5. Conclusions

In summary, the high-molecular-weight α-d-Glucan MP-A, isolated from the hard-shelled mussel (*Mytilus coruscus*), has been observed to exert an anti-inflammatory effect, and thus represents a promising therapeutic candidate for the control of disease-associated pro-inflammatory mediator overproduction. The current study provides a preliminary pharmacological basis, and underlying molecular mechanism, for the use of MP-A in the control of inflammatory disorders.

## Abbreviations

LPS	lipopolysaccharide
TLR4	Toll-like receptor 4
TLR2	Toll-like receptor 2
TLRs	toll like receptors
TNF-α	tumor necrosis factor α
iNOS	inducible nitric oxide synthase
COX-2	cyclooxygenase-2
PGE_2_	prostaglandin E2
SPR	surface plasmon resonance
CCK-8	Cell Counting Kit-8
PMA	phorbol-12-myristate-13-acetate
DAPI	2-(4-amidinophenyl)-6-indolecarbamidine dihydrochloride
MAPK	mitogen-activated protein kinase
ERK	extracellular signal regulated kinase
JNK	c-Jun N terminal kinase
NF-κB	nuclear factor-kappa B

## References

[1] Xu H., Guo T., Guo Y.F., Zhang J., Li Y., Feng W., Jiao B. (2008). Characterization and protection on acute liver injury of a polysaccharide mp-i from *mytilus coruscus*. Glycobiology.

[2] Kim E.-K., Oh H.-J., Kim Y.-S., Hwang J.-W., Ahn C.-B., Lee J.S., Jeon Y.-J., Moon S.-H., Sung S.H., Jeon B.-T. (2013). Purification of a novel peptide derived from *mytilus coruscus* and in vitro/in vivo evaluation of its bioactive properties. Fish Shellfish Immunol..

[3] Kim Y.S., Ahn C.B., Je J.Y. (2016). Anti-inflammatory action of high molecular weight mytilus edulis hydrolysates fraction in lps-induced raw264.7 macrophage via nf-kappab and mapk pathways. Food Chem..

[4] Li G., Fu Y., Zheng J., Li D. (2014). Anti-inflammatory activity and mechanism of a lipid extract from hard-shelled mussel (*mytilus coruscus*) on chronic arthritis in rats. Mar. Drugs.

[5] Tzianabos A.O. (2000). Polysaccharide immunomodulators as therapeutic agents: Structural aspects and biologic function. Clin. Microbiol. Rev..

[6] Cavaillon J.M. (1994). Cytokines and macrophages. Biomed. Pharmacother..

[7] Miller T.E., Ormrod D.J., Geddes R. (1993). Anti-inflammatory activity of glycogen extracted from perna canaliculus (nz green-lipped mussel). Agents Actions.

[8] Cesaretti M., Luppi E., Maccari F., Volpi N. (2004). Isolation and characterization of a heparin with high anticoagulant activity from the clam tapes phylippinarum: Evidence for the presence of a high content of antithrombin iii binding site. Glycobiology.

[9] Luan J., Xin T., Chu Z.Y., Wang J.C., Zhang S., Shen Y. (2010). Immunomodulation of mytihus edulis linnaeus polysaccharides in mice. Chin. J. Mar. Drugs.

[10] Mao J., Hou Z., Chen D., Zhang W. (2014). Anti-hyperlipi-demic effects of polysaccharide ma from *mytilus coruscus* on experimental atherosclerosis in rabitts. J. Pharm. Pract..

[11] Czop J.K. (1986). The role of beta-glucan receptors on blood and tissue leukocytes in phagocytosis and metabolic activation. Pathol. Immunopathol. Res..

[12] Williams D.L. (1997). Overview of (1→3)-β-d-glucan immunobiology. Mediat. Inflamm..

[13] Ross G.D., Vetvicka V., Yan J., Xia Y., Vetvickova J. (1999). Therapeutic intervention with complement and beta-glucan in cancer. Immunopharmacology.

[14] Muller A., Rice P.J., Ensley H.E., Coogan P.S., Kalbfleish J.H., Kelley J.L., Love E.J., Portera C.A., Ha T., Browder L.W. (1996). Receptor binding and internalization of a water-soluble (1→3)-β-d-glucan biologic response modifier in two monocyte/macrophage cell lines. J. Immunol..

[15] Xu X., Yasuda M., Nakamura-Tsuruta S., Mizuno M., Ashida H. (2012). Beta-glucan from lentinus edodes inhibits nitric oxide and tumor necrosis factor-alpha production and phosphorylation of mitogen-activated protein kinases in lipopolysaccharide-stimulated murine raw 264.7 macrophages. J. Biol. Chem..

[16] Yang X.B., Gao X.D., Han F., Xu B.S., Song Y.C., Tan R.X. (2005). Purification, characterization and enzymatic degradation of ycp, a polysaccharide from marine filamentous fungus phoma herbarum ys4108. Biochimie.

[17] Zhu R., Zhang X., Liu W., Zhou Y., Ding R., Yao W., Gao X. (2014). Preparation and immunomodulating activities of a library of low-molecular-weight alpha-glucans. Carbohydr. Polym..

[18] Chanput W., Mes J.J., Wichers H.J. (2014). Thp-1 cell line: An in vitro cell model for immune modulation approach. Int. Immunopharmacol..

[19] Beutler B., Rietschel E.T. (2003). Innate immune sensing and its roots: The story of endotoxin. Nat. Rev. Immunol..

[20] Lee S.H., Soyoola E., Chanmugam P., Hart S., Sun W., Zhong H., Liou S., Simmons D., Hwang D. (1992). Selective expression of mitogen-inducible cyclooxygenase in macrophages stimulated with lipopolysaccharide. J. Biol. Chem..

[21] Buchanan M.M., Hutchinson M., Watkins L., Yin H. (2010). Toll-like receptor 4 in cns pathologies. J. Neurochem..

[22] Uto T., Fujii M., Hou D.-X. (2005). 6-(methylsulfinyl)hexyl isothiocyanate suppresses inducible nitric oxide synthase expression through the inhibition of janus kinase 2-mediated jnk pathway in lipopolysaccharide-activated murine macrophages. Biochem. Pharmacol..

[23] Zar P.P.K., Morishita A., Hashimoto F., Sakao K., Fujii M., Wada K., Hou D.-X. (2014). Anti-inflammatory effects and molecular mechanisms of loquat (eriobotrya japonica) tea. J. Funct. Foods.

[24] Zhou J., Zhu P., Jiang J.L., Zhang Q., Wu Z.B., Yao X.Y., Tang H., Lu N., Yang Y., Chen Z.N. (2005). Involvement of cd147 in overexpression of mmp-2 and mmp-9 and enhancement of invasive potential of pma-differentiated thp-1. BMC Cell Biol..

[25] Zhuang X., Pang X., Zhang W., Wu W., Zhao J., Yang H., Qu W. (2012). Effects of zinc and manganese on advanced glycation end products (ages) formation and ages-mediated endothelial cell dysfunction. Life Sci..

[26] Xu Z., Lin S., Wu W., Tan H., Wang Z., Cheng C., Lu L., Zhang X. (2008). Ghrelin prevents doxorubicin-induced cardiotoxicity through tnf-alpha/nf-kappab pathways and mitochondrial protective mechanisms. Toxicology.

[27] Wonderling R.S., Ghaffar A., Mayer E.P. (1996). Lipopolysaccharide-induced suppression of phagocytosis: Effects on the phagocytic machinery. Immunopharmacol. Immunotoxicol..

[28] De Lima T.M., Sampaio S.C., Petroni R., Brigatte P., Velasco I.T., Soriano F.G. (2014). Phagocytic activity of lps tolerant macrophages. Mol. Immunol..

[29] Li P., Zhang X., Cheng Y., Li J., Xiao Y., Zhang Q., Zong A., Zhong C., Wang F. (2014). Preparation and in vitro immunomodulatory effect of curdlan sulfate. Carbohydr. Polym..

[30] Liu F., Zhang X., Ling P., Liao J., Zhao M., Mei L., Shao H., Jiang P., Song Z., Chen Q. (2017). Immunomodulatory effects of xanthan gum in lps-stimulated raw 264.7 macrophages. Carbohydr. Polym..

[31] Davis K.L., Martin E., Turko I.V., Murad F. (2001). Novel effects of nitric oxide. Annu. Rev. Pharmacol. Toxicol..

[32] Nathan C. (1992). Nitric oxide as a secretory product of mammalian cells. FASEB J..

[33] Ohno N., Hashimoto T., Adachi Y., Yadomae T. (1996). Corrigendum to: “Conformation dependency of nitric oxide synthesis of murine peritoneal macrophages by β-glucans in vitro” [immunol. Lett. 52 (1996) 1–7]. Immunol. Lett..

[34] Gupta S.C., Sundaram C., Reuter S., Aggarwal B.B. (2010). Inhibiting nf-kappab activation by small molecules as a therapeutic strategy. Biochim. Biophys. Acta.

[35] Iezzi A., Ferri C., Mezzetti A., Cipollone F. (2007). Cox-2: Friend or foe?. Curr. Pharm. Des..

[36] Han G., Wang G., Zhu X., Shao H., Liu F., Yang P., Ying Y., Wang F., Ling P. (2012). Preparation of xanthan gum injection and its protective effect on articular cartilage in the development of osteoarthritis. Carbohydr. Polym..

[37] Kim Y.S., Ko H.M., Kang N.I., Song C.H., Zhang X., Chung W.C., Kim J.H., Choi I.H., Park Y.M., Kim G.Y. (2007). Mast cells play a key role in the developmentof late airway hyperresponsiveness through tnf-αin a murine model of asthma. Eur. J. Immunol..

[38] Roux C.H., Brocq O., Breuil V., Albert C., Euller-Ziegler L. (2006). Safety of anti-tnf-alpha therapy in rheumatoid arthritis and spondylarthropathies with concurrent b or c chronic hepatitis. Rheumatology.

[39] Stokkers P., Camoglio L., Van Deventer S. (1994). Tumor necrosis factor (tnf) in inflammatory bowel disease: Gene polymorphisms, animal models, and potential for anti-tnf therapy. J. Inflamm..

[40] Brown G.D. (2006). Dectin-1: A signalling non-tlr pattern-recognition receptor. Nat. Rev. Immunol..

[41] Gantner B.N., Simmons R.M., Canavera S.J., Akira S., Underhill D.M. (2003). Collaborative induction of inflammatory responses by dectin-1 and toll-like receptor 2. J. Exp. Med..

[42] Takeuchi A., Kamiryou Y., Yamada H., Eto M., Shibata K., Haruna K., Naito S., Yoshikai Y. (2009). Oral administration of xanthan gum enhances antitumor activity through toll-like receptor 4. Int. Immunopharmacol..

[43] Shimazu R., Akashi S., Ogata H., Nagai Y., Fukudome K., Miyake K., Kimoto M. (1999). Md-2, a molecule that confers lipopolysaccharide responsiveness on toll-like receptor 4. J. Exp. Med..

[44] Fujimoto T., Yamazaki S., Eto-Kimura A., Takeshige K., Muta T. (2004). The amino-terminal region of toll-like receptor 4 is essential for binding to md-2 and receptor translocation to the cell surface. J. Biol. Chem..

[45] Moynagh P.N. (2005). The nf-κb pathway. J. Cell Sci..

[46] Tak P.P., Firestein G.S. (2001). Nf-κb: A key role in inflammatory diseases. J. Clin. Investig..

